# Tug-of-War Injuries: A Case Report and Review of the Literature

**DOI:** 10.1155/2014/519819

**Published:** 2014-10-28

**Authors:** Pranit N. Chotai, Amr A. Abdelgawad

**Affiliations:** Department of Orthopedic Surgery, Texas Tech University Health Sciences Center, El Paso, TX 79905, USA

## Abstract

We report a case of a 10-year-old boy presenting with radial nerve palsy due to injury during a tug-of-war game. Patient was managed nonoperatively and regained radial nerve function. Tug-of-war is a globally popular noncontact sport. Injuries during this game are inevitable and may range from simple sprains to life and limb threatening trauma. Combined hip and knee injuries and soft-tissue injuries involving the back are most frequent. Most injuries occur when tug-of-war was played in an informal setting and where the tug-of-war International Federation rules were less likely to be followed. Measures should be taken to increase the awareness about these safety rules and prevention of consequent injuries. Sports physicians, pediatricians, orthopedic surgeons, general physicians, and athletic trainers should be aware of potential injuries resulting from this game while caring for these athletes, so as to be well prepared for apt management of the injuries associated with TOW.

## 1. Introduction

Tug-of-war (TOW) is a globally played sport and involves professional and amateur athletes from both genders and almost all age groups [1, 2]. Typically, two teams of “pullers” participate and apply enormous contradirectional forces on the pulling rope. Like any other sport, injuries during this game are inevitable. However, there is scarcity of medical literature discussing patterns and biomechanics of injuries sustained during TOW. Here, we report a case of radial nerve palsy in a child who was playing TOW recreationally. Review of literature for all other TOW-related injuries reported to date is also presented. To the best of our knowledge, no such review focusing on injuries related to this popular sport has been performed earlier.

## 2. Case Report

**Figure 1:**
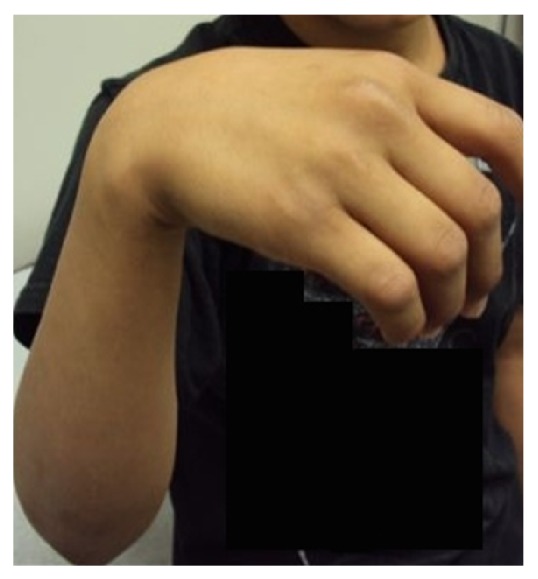
Clinical photograph showing a complete right wrist drop at presentation.

**Figure 2:**
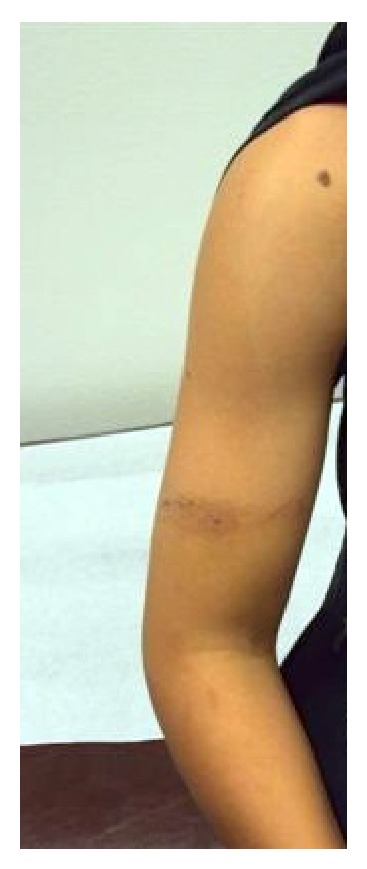
Contusion mark on the right arm due to tight twisting of rope during the tug-of-war game.

A 10-year-old right-handed boy was brought in by his mother for complaint of weakness of right forearm and wrist immediately after participating in a recreational tug-of-war game. There was no history of direct trauma or fall; however, the patient did report forceful pulling of the TOW rope with three twists applied to his right upper arm. On examination, his vitals were stable and radial pulse was palpable; however, he was unable to actively extend the right wrist (Figure 1). There were no open wounds on the right upper extremity; however, there was a circumferential contusion mark on the lower third of the right upper arm (Figure 2). Systemic examination revealed no additional findings. Patient was put in a functional wrist brace and was started on nonsteroidal anti-inflammatory drug with physical therapy. At 2 months' follow-up, radial nerve function improved and at 3 months' follow-up, the patient regained complete wrist function.

## 3. Discussion

### 3.1. History of Tug-of-War

TOW has been first described as early as 4000 years ago as a ropeless version in the artwork on Egyptian tomb engravings. The origin of the sport has also been described in histories from countries like India, China, Korea, France, Scandinavia, Great Britain, and South America. Towards the end of 19th century, TOW became an organized sport in parts of Europe and it was included in the Olympic Games held through 1900 to 1920. In subsequent years, various TOW associations were formed to resurrect the game. The TOW Association (ToWA) was formed in England in 1958 and TOW International Federation (TWIF), which regulates global practice of the game, was formed in 1960. In 1978, the United States Amateur TOW Association (USATOWA) was formed and in 1981 TOW was included for the first time in World Games held in the US. Since then, it has been a popular sport all over the world, recreationally as well as professionally.

In its contemporary form, TOW is played as per the rules laid out by TWIF which has 59 member countries and administrative headquarters in the United States [4]. It is organized as indoor or outdoor event involving two teams with 8 players (or “pullers”) each, pulling against opponent team on a rope. Teams are categorized based on weight classes ranging from 480 to 720 kilograms per team and also as men, women, senior (>18 years old for males and >16 years old for females), under 23, and junior (15–18 years old) teams [4]. There is a considerable overlap in categorization according to weight and age, and this allows athletes to participate across multiple teams. A best of three pulls is the typical format with a mean duration of each pull of 2 minutes 30 seconds with a rest period of six minutes in between the pulls [5].

### 3.2. Injuries during TOW Game

Although TOW is a noncontact sport, the associated injuries are unavoidable due to the high amount of energy involved in pulling the rope. A recent study reports that TOW athletes can generate force equal to 149% of weight in dynamic pull and 200% of weight in static pull, which is considerably high [18]. Our literature search for reports discussing injuries related to participation in a TOW game revealed two large studies and fourteen case reports (Table 1). A questionnaire based retrospective survey was performed on a cohort of 990 pullers from 22 countries participating in the 1998 World TOW Championship held in Rochester, MN, USA [1]. The participants were given a tug-of-war injury survey (TWIS) questionnaire to self-report injury data from TOW-related injuries. However, only 252 athletes participated and since there was no comparison group, validity testing of this data was not possible. The response rate from male and female athletes was similar (26% males versus 25% females) [1]. Another study is the TWIF Epidemiology of Injury Report released in 2003. This is a prospective study monitoring 544 participants from 68 teams during the World Outdoor TOW Championship, 2002 [3]. It has not been formally published in medical literature and instead it is available on TWIF website (http://www.tugofwar-twif.org/). In the TWIF research report, the injury was defined as one which required complete cessation of participation and/or which required medical treatment during the match, whereas the definition of injury in the prior retrospective study was broad. Additionally, the TWIF report analyses the injuries in relation to exposure time and expresses injury incidence per puller-hours. It also analyses the injury based on the TWIF participant categories. Apart from these two studies, all other TOW-related injuries are reported as isolated cases [6–9, 11–13, 15–17].

Based on the currently available TOW-related injury reports, the injuries can be classified as follows.

**Figure 3:**
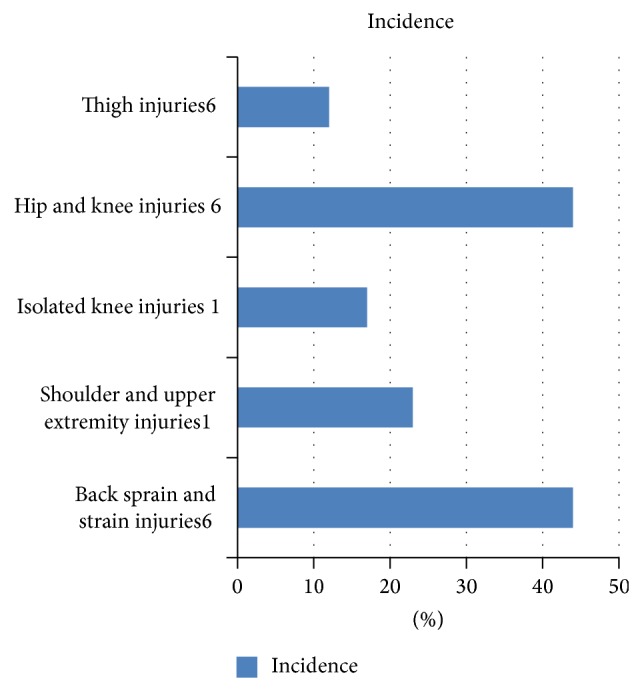
Common types of tug-of-war injuries [1, 3].

*(A) Back Sprain and Strain Injuries*. Enormous stresses are exerted on the back and spine due to excessive spinal flexion and due to force passing through the axial skeleton during the active “pull” phase of the game. Correspondingly, the incidence of back injuries was as high as 42%–44% (Figure 3) in both large studies [1, 3]. In a study determining physiological and metabolic characteristics of elite TOW athletes it was found that these athletes have excellent strength and endurance relative to body size but relatively low explosive leg power and back flexibility [5]. This may also explain the high likelihood of sustaining back injuries in TOW.

*(B) Joint and Extremity Injuries*. Major joints and extremity injuries are the second most common injuries following back injuries in TOW athletes. Smith and Krabak found that combined shoulder and elbow injuries accounted for 23% injuries and 17% injuries (Figure 3) involved the knee joint in their study [1]. Similarly, TWIF research report reveals 44% injuries to hip and knee combined and 12% injuries to thigh. Most of these injuries were muscle strain injuries (78%) and a few presented as joint sprains [3]. However, the exact nature of individual injuries is not described in detail in these two large studies. Isolated injuries to extremities ranging from irreducible elbow dislocation to cases with traumatic amputations of forearm, thumb, and fingers during recreational TOW games have also described as case reports [6, 7, 12–14]. In a massive TOW game organized in an informal setting in Taiwan, 1500 people participated of which 42 sustained injuries following the fall when the rope broke apart [13, 15]. Two patients sustained type III traction avulsion transforearm amputation, one sustained a wrist fracture, and one sustained right hand amputation [15]. It is believed that this devastating injury was not due to the force exerted to pull the rope and instead it resulted due to the rebound force generated when the rope ruptured. Notably, the rope used in this event was unusual and had a central trunk with periodic branches, which were pulled by the participants. Both patients with transforearm amputation underwent replantation with delayed nerve grafting and regained hand functions [13]. Similarly, an 11-year-old Australian boy suffered right palm and finger amputation resulting from to-and-fro movement of the rope which was wrapped around the fingers of his right hand. The hand anatomy and function were restored by microsurgical repair of the amputation [14]. A case of a 3-year-old child who developed volar forearm compartment syndrome has also been described [16]. At presentation, it was believed that the rise in forearm compartment pressures was due to the fall while playing, but it was later attributed to traction injury sustained by flexor muscles in the forearm while playing the TOW. A case with biceps tendon rupture following TOW game is described. This patient sustained a power chain saw induced brachial plexus injury which was repaired surgically. After regaining the biceps strength, he participated in TOW game 3 years later when he ruptured the biceps tendon [10]. All upper extremity injuries seem to be a direct or indirect result of undue stress put on the forearm muscles resulting from pulling the rope. The force generated by twisting of the rope around the extremity acts in a circumferential manner to induce long bone fractures and then leads to avulsion amputation of the extremity [12]. Our patient did not present with a dramatic injury; however, he presented with a complete wrist drop which recovered fully over 3 months of conservative treatment. Lower extremity plays a dynamic role in linking the rope with the ground and contributes effectively to the “pull”; however we did not find any reports describing a lower limb injury related to TOW participation other than the simple sprain and strain injuries described in two large studies.

*(C) Visceral Injuries*. During the same massive TOW game described earlier, a participant sustained multiple visceral injuries with multilevel cervical intervertebral disc herniation and corresponding spinal cord injury. It was believed that the visceral injuries in this patient were a result of stepping after the rope broke and the spinal cord injury may also have resulted from hyperextension of the neck. He was treated with an open repair of liver and spleen rupture and discectomy for prolapsed intervertebral discs and neurolysis for brachial plexus injury. At 2 years' follow-up this patient is dependent on assistive devices for activities of daily living [15]. In a retrospective study determining the characteristics of sport- and recreation-related spinal cord injuries, a single case with T10 spinal cord injury attributable to TOW participation was also described [17]. A case with extensive retinal hemorrhage and one with Bochdalek hernia following TOW game have been described; both of these injuries are attributed to Valsalva effect during the pull [9, 11].

*(D) Rib and Thoracic Cage Injuries*. It was shown that the game of rowing and TOW apparently place similar stresses on trunk musculature [19]. Although no report was found actually mentioning rib or thoracic cage injury, according to Smith and Krabak, those who reported “side” and “back” injuries in their study may have suffered from rib and thoracic cage injuries [1].

*(E) Injuries to Skin*. In the Smith and Krabak study, abrasion, burn, and blister of the skin represented 41% of total injuries with additional 12% injuries described as bruise/contusion. In particular, blisters of hand and unilateral rib scarring have been observed by previous authors [1]. Our patient did present with bruising of arm due to compression injury following twisting of the rope around the arm.

### 3.3. Factors Affecting Incidence of Injuries and Prevention of Injuries

Numerous factors seem to increase the potential for sustaining injury in TOW athletes. Although the injuries are inevitable, they can definitely be prevented. On careful observation, it was found that majority of TOW-related injuries resulted when the game was played in a casual or recreational setting compared to formal athletic events, where TWIF rules were more likely observed. We concur with the previous authors, who suggested that TWIF rules (available at http://www.tugofwar-twif.org/) should be strictly adhered to, to minimize the risk of injuries [15]. Additionally, TWIF guidance booklet on training and coaching the junior participants provides valuable information which can help decrease the incidence of injuries in school participants (http://www.tugofwar-twif.org/). The following factors may play a role in decreasing the risk of injuries in TOW participants.

*Age*. The youngest patient presenting with TOW-related injury was 3 years old and the eldest patient was 64 years old in this review [15, 16]. No age group is immune to injuries; however, apparently severe injuries were commonly found in younger participants (Table 1). In a TWIF research report focusing on participants in the “youth” category, it was noted that there is an increased possibility of acute avulsion injury to the epiphyseal plate as the ligaments in a child are 2–5 times stronger than the cartilage and bone compared to that of an adult [2].

*Gender*. In the previous studies, although the injury patterns of male and female pullers were similar, it was found that female athletes had an increased injury rate compared to male participants; however this finding did not reach statistical significance and needs further research [1, 3]. Since the enormous pulling forces are inherent to the game, participant categorization based on gender and weight classes is necessary to reduce the risk of potential injuries during this game [2].

*Number of Pullers*. Typically eight pullers participate on each team and a minimum of seven pullers are mandatory to continue the game. In majority of cases, including our case (6 players on each team), the number of pullers on each side was less or in excess of the stipulated number on each side [4].

*Position of the Puller.* The TWIS questionnaire did not collect information regarding the position of the athlete and its relation to injuries; however, the TWIF research study found that 32% of injuries were seen in participants at back of the rope (position 6) and 12% injuries were seen in anchors (position 8) and 12% injuries in those at position 3. Our patient and the child with finger amputation were at position one and wrapped the rope around their arm and fingers, respectively [3].

### 3.4. TWIF Rules Pertaining to Safety, Protective Gears, and Equipment

As per TWIF manual, the rope must not be less than 10 centimeters or more than 12.5 centimeters in circumference and should have a minimum length of 33.5 meters. For younger participants, a thinner rope with reduced pulling distance is recommended [2]. No knots or loops are allowed on the rope and it shall not be locked across any part of the body of any participant. Only the participant at anchor position is allowed to pass the rope diagonally across the back and over the opposite shoulder from rear to front and pass the remaining rope under the armpit in a backward and outward direction. For all pullers, the rope grip should be “Ordinary” with palms of both hands facing up and with both arms extended forward. An anchor vest with a maximum thickness of 5 centimeters must always be worn under sport clothing and placed between the rope and body. No constructions intended to lock the rope are allowed under the protective clothing. Shoes used by “pullers” should be devoid of any metal, spikes, or nails and the sole and heel should perfectly flush [4].

## 4. Conclusion

Tug-of-war is an athletic and recreational noncontact sport with increasing popularity. Injuries during this game are inevitable and underreported. The spectrum of injuries may range from simple sprains to life and limb threatening complications. Depending on the severity of injuries, they may be managed nonoperatively or surgically. Additionally, orthopedic surgeons, sports physicians, pediatricians, athletic trainers, and general physicians should be aware of potential injuries resulting from this game so as to be well-prepared for better management of the injuries associated with the tug-of-war.

## Figures and Tables

**Table 1a:** (a) Clinical studies

	Reference, publication	Nature of TOW game	Nature of injury or presentation	Management and outcome	Comments
1	Smith and Krabak, Scand. J. Sports Med., 2002 [1]	Professional: World TOW Championship 1998, Rochester, MN	149 injuries: 42% back injuries 23% shoulder + upper extremity 17% knee injuries	NA	Questionnaire survey study. 252 of 990 (25%) pullers completed the survey

2	Van-Heerden and Van-Rensburg, TWIF Research Report Project 1, 2003 [3]	Professional: World Outdoor Championship 2002	Total 9 injuries: 44% injuries to lower back 44% to hip and knee and 12% to thigh	67% did not return to sport	Report from a cohort of 544 subjects from 68 teams

**Table 1b:** (b) Cases or case reports.

	Reference, publication	Nature of TOW game	Nature of injury or presentation	Management and outcome	Comments
1	Pawlowski et al. [6], J. Trauma 1970 17 y/F	? Recreational	Irreducible posterolateral dislocation of elbow joint	Open reduction and repair of joint ligaments and capsule. Returned to full activities at 11th week	NA

2	Pedersen and Holset. [7], Tandlaegebladet. 1981 [Danish]	NA	Thumb amputation	NA	NA

3	Ferguson and Kierkegaard [8], Ugeskr Laeger. 1981 [Danish]	NA	Trauma resulting from tug-of-war	NA	NA

4	Morán [9], Cesk Oftalmol. 1984 [Article in Czech]	NA	Extensive retinal hemorrhage	NA	Injury was believed to result from the Valsalva effect during the pull

5	Demuynck and Zuker [10], Acta Orthop. Be. 1995 16 y/M	NA	Biceps tendon rupture. Past history of microneural repair of left brachial plexus injury	Muscle strengthening physiotherapy	Injury attributed to strength regained following musculocutaneous nerve repair

6	Iiai et al. [11], Kyobu Geka. 1997 [Article in Japanese] 38 y/F	Formal: athletic meeting	Bochdalek hernia with incarceration of transverse colon in left thoracic cavity	Hernia repair with direct suturing through a thoracotomy	Injury was believed to result from the Valsalva effect during the pull

7	Bruce and Hayes [12], Orthopedics 1999 21 y/M	Informal: 25 pullers per team	Right dominant—transforearm amputation with multilevel avulsion of tendons, arteries, and nerves	Mid forearm amputation. Returned to full activity at 8 weeks post-op with below-elbow prosthesis	Patient was in the front and looped rope around distal forearm 2 inches proximal to wrist joint

8	Chuang et al. [13], Plast. and Recon. Surg. 2001	Casual—massive TOW game	2 patients with type III traction avulsion transforearm amputation	Replantation with delayed nerve grafting at 6 months after injury	Rope had central trunk and multiple branches and ruptured at mid-point of central trunk

9	LACUNAE [14] 2002 11 y/M	Recreational—supervised schoolyard game	Right palm—finger amputation	Microsurgery to restore the hand with good prognosis	Patient was at the front of his team and wrapped rope around the right fingers

10	Lin et al. [15], J. Chin. Med. Assoc. 2003 64 y/M	Casual—massive TOW game	Multiple injuries: liver and spleen rupture, bilateral brachial plexus injury, and spinal cord injury—C5-6 intervertebral disc herniation	Surgical repair of injuries with discectomy and neurolysis. At 2 years, ADL was partially restored with assistive devices	Rope had central trunk and multiple branches and ruptured at mid-point of central trunk

11	Choi et al. [16], Am. J. Orthop. 2008 3 y/F	Casual: played TOW on bed and fell down and injured the forearm	Volar forearm compartment syndrome	Volar compartment release and surgical exploration. Full restoration of hand function at 3 months	Rupture of flexor digitorum profundus muscle due to traction injury during TOW

12	Ye et al. [17], Spinal Cord 2009	? Recreational	Single patient with a T10 spinal cord injury	NA	NA

13	Current case—2012 10 y/M	Recreational school picnic game	High radial nerve palsy presented as immediate weakness of forearm extensor muscles and wrist drop	Conservative treatment with wrist brace. Complete recovery at 3 months follow-up	The patient was in position one in a team of 6 and looped the rope 3 turns around the right upper arm

## References

[1] Smith J., Krabak B. (2002). Tug of war: introduction to the sport and an epidemiological injury study among elite pullers. *Scandinavian Journal of Medicine and Science in Sports*.

[2] van-Heerden H. J., Rossouw F., Ball S., Kuisis S. (2003). *Youth Focus. World Outdoor Championships 2002 Tug-of-War Medical Research Report Project-6*.

[3] Van-Heerden H. J., Van-Rensburg C. (2003). *Tug of War International Federation: World Outdoor Championships 2002 : Report Project-1 Epidemiology of Injury*.

[4] http://www.tugofwar-twif.org/?p=rules&id=14&nav=1.

[5] Warrington G., Ryan C., Murray F., Duffy P., Kirwan J. P. (2001). Physiological and metabolic characteristics of elite tug of war athletes. *British Journal of Sports Medicine*.

[6] Pawlowski R. F., Palumbo F. C., Callahan J. J. (1970). Irreducible posterolateral elbow dislocation: report of a rare case.. *Journal of Trauma*.

[7] Pedersen S. S., Holst E. (1981). Tug o'war—a dangerous sport. *Tandlaegebladet*.

[8] Ferguson A., Kierkegaard A. (1981). Traumata resulting from tug-of-war. *Ugeskrift for Laeger*.

[9] Morán M. (1984). Extensive retinal hemorrhage after a game of tug-of-war (Valsalva's hemorrhagic retinopathy). *Ceskoslovenska Oftalmologie*.

[10] Demuynck M., Zuker R. M. (1995). Biceps tendon rupture after successful reinnervation: a case report. *Acta Orthopaedica Belgica*.

[11] Iiai T., Ohmori K., Ohtaki M., Mishina T., Saitoh H., Ishihara R., Suzuki N. (1997). Adult bochdalek hernia after playing at a tug of war. *The Japanese Journal of Thoracic Surgery*.

[12] Bruce W. D., Hayes C. W. (1999). 'Tug-of-war hand': transforearm amputation by an unusual mechanism. *Orthopedics*.

[13] Chuang D. C.-C., Lai J.-B., Cheng S.-L., Jain V., Lin C.-H., Chen H.-C. (2001). Traction avulsion amputation of the major upper limb: a proposed new classification, guidelines for acute management, and strategies for secondary reconstruction. *Plastic and Reconstructive Surgery*.

[14] (2002). LACUNAE. *Injury Prevention*.

[15] Lin P.-H., Chiu F.-Y., Hsiao N.-C., Chuang T.-Y. (2003). Injuries during a massive tug-of-war game. *Journal of the Chinese Medical Association*.

[16] Choi G., Huang J. L., Fowble V., Tucci J. (2008). Volar forearm compartment syndrome following flexor digitorum profundus muscle rupture in a 3-year-old girl. *American Journal of Orthopedics*.

[17] Ye C., Sun T., Li J., Zhang F. (2009). Pattern of sports-and recreation-related spinal cord injuries in Beijing. *Spinal Cord*.

[18] Tanaka K., Ushizu A., Minamitani N., Fukushima M., Yamamoto H. Biomechanical analysis on dynamic pulling skill for elite indoor tug of war athletes. https://ojs.ub.uni-konstanz.de/cpa/article/view/774.

[19] Karlson K. A. (1998). Rib stress fractures in elite rowers. A case series and proposed mechanism. *The American Journal of Sports Medicine*.

